# Invasion dynamics and ecological impacts of *Anisakis typica* in commercial fish from the Western Pacific Ocean

**DOI:** 10.14202/vetworld.2025.1365-1376

**Published:** 2025-05-31

**Authors:** Dhito Dwi Pramardika, Fadjar Satrija, Sulistiono Sulistiono, Risa Tiuria, Arifin Budiman Nugraha

**Affiliations:** 1 Veterinary Biomedical Sciences Study Program, Graduate School of IPB University, Bogor, 16680, Indonesia; 2 Department of Health, Nusa Utara State Polytechnic, Sangihe Islands, North Sulawesi 95812, Indonesia; 3 Division of Parasitology and Medical Entomology, School of Veterinary Medicine and Biomedical Sciences, IPB University, Bogor, 16680, Indonesia; 4 Department of Aquatic Resources Management, Faculty of Fisheries and Marine Sciences, IPB University, Bogor, 16680, Indonesia

**Keywords:** *Anisakis typica*, fisheries sustainability, marine fish, parasitology, Western Pacific Ocean, zoonosis

## Abstract

**Background and Aim:**

The Western Pacific Ocean hosts rich marine biodiversity, yet the parasitic infections affecting its commercial fish species remain underexplored. This study investigated the prevalence, intensity, morphological characteristics, molecular identity, and ecological impacts of *Anisakis typica* infection in commercial fish from this region.

**Materials and Methods:**

Between January and April 2024, 178 fish samples from 12 commercial species were collected across three geographical clusters: Sangihe Island, Kahakitang Island, and Marore Island. Fish specimens were morphologically identified and dissected for parasite detection. Morphological identification of larvae was complemented by molecular characterization through polymerase chain reaction amplification of the internal transcribed spacer (ITS)1–5.8S–ITS2 rDNA region, followed by sequencing and phylogenetic analysis.

**Results:**

*A. typica* infection was observed in 10.7% of the fish sampled, with moderate infection intensity. *Euthynnus affinis* exhibited the highest prevalence (41.2%) and intensity (15.4). The intestinal wall was the dominant predilection site (96.3%). Molecular analyses confirmed *A. typica* infection in *E. affinis*, *Katsuwonus pelamis*, *Decapterus kurroides*, and *Variola louti*, with high genetic similarity (93.38%–100%) to isolates previously reported from China. Notably, this study provides the first documentation of *A. typica* infection in *D. kurroides* and *V. louti*.

**Conclusion:**

This study highlights the ecological and zoonotic significance of *A. typica* infection in commercially important fish from the Western Pacific Ocean. The findings underscore the potential threats to marine ecosystem stability, fishery sustainability, and public health. High genetic proximity between *A. typica* isolates from Indonesia and China suggests historical host migrations, emphasizing the need for regional surveillance and integrated management strategies. Enhanced inspection practices and public awareness initiatives are crucial to mitigate the zoonotic risks posed by consuming infected fish.

## INTRODUCTION

The fisheries sector significantly contributes to food security and economic growth in archipelagic nations such as Indonesia, China, and India, which account for the highest marine capture volumes globally [1]. In 2021, Indonesian fishing companies produced 110,246 tons of fish, corresponding to a market value of USD 156 billion. The capture fisheries sector generates more revenue than aquaculture, producing a variety of species including skipjack, tuna, shrimp, and spiny dogfish [2].

Food safety remains a major concern within Indonesia’s fishery industry. The substantial production of diverse fish species increases the vulnerability to parasitic contamination, posing notable public health risks. Inadequate fish quality control further exacerbates this issue. Consumption of fish contaminated with parasites such as *Anisakis* spp. can lead to anisakiasis, characterized by symptoms including abdominal pain, nausea, vomiting, abdominal distension, fever, persistent diarrhea with bleeding, anemia, and respiratory distress [3, 4]. *Anisakis* spp., members of the *Anisakidae* family, are nematodes capable of infecting fish, birds, reptiles, mammals, and humans [5]. This zoonotic, foodborne disease arises from the ingestion of raw or undercooked fish harboring *Anisakidae* larvae, notably species of *Anisakis*, *Pseudoterranova*, and *Contracaecum* [6].

Beyond gastrointestinal manifestations, *Anisakis* spp. are implicated in allergic reactions in humans, due to allergens such as Ani s1, Ani s4, Ani s5, and Ani s7, which persist even after the larvae are killed during cooking [7]. Allergic responses can range from hives, respiratory difficulties, and rashes to severe conditions such as anaphylaxis and acute urticaria [3].

Globally, *Anisakis* spp. infections are widespread across marine environments. The reported prevalence rates are high, reaching 75% in *Scomber japonicus* and 76.8% in skipjack tuna [8, 9]. In the Philippines, the prevalence of *Decapterus* species was recorded at 22%. Although *Anisakis* spp. infections have been reported in various marine fish species across Indonesia, molecular identification studies remain limited, and prevalence data from regions such as North Sulawesi, particularly Sangihe Island Regency, are lacking.

The high burden of *Anisakis* spp. infection also adversely affects fish populations, as larvae migrate from the digestive tract to the body cavity [10]. Effective control measures are essential to maintain the integrity of the fish supply chain and protect public health. Species-specific identification of both fish hosts and infecting *Anisakidae* parasites is necessary to inform the public about species at higher risk of infection.

The Sangihe Islands Regency, located adjacent to the Philippines, features unique oceanographic conditions characterized by southward currents toward the Indian Ocean and northward flows through the Mindanao and Kuroshio currents [11]. These conditions influence fish migration patterns and facilitate the dissemination of *Anisakis* species [12]. Fish caught in this region are primarily consumed locally, while a portion is exported to major destinations including Malaysia, Singapore, Japan, Taiwan, and China [13].

Despite numerous reports of *Anisakis* spp. infection in various commercial fish species across Indonesia, there remains a significant paucity of molecular-level identification studies, particularly in the waters surrounding North Sulawesi, including the Sangihe Islands Regency. Most existing investigations have been limited to morphological assessments, offering insufficient taxonomic resolution to distinguish closely related *Anisakis* species. Moreover, regional variations in the prevalence, intensity, and distribution of *Anisakis* spp. infections remain poorly characterized, despite the area’s unique oceanographic conditions that may influence parasite dissemination. Critically, no prior studies have molecularly confirmed the presence and genetic diversity of *Anisakis typica* in commercial fish from the Indonesian–Philippine maritime border, an area that serves both domestic consumption and international export markets. This lack of precise epidemiological and molecular data hinders the development of effective public health interventions and fisheries management strategies, particularly given the zoonotic potential and allergenic risks associated with *Anisakis* infections.

This study aimed to address these knowledge gaps by systematically investigating the invasion dynamics and ecological impacts of *A. typica* infection in commercially important fish species from the Western Pacific Ocean, focusing on the Sangihe Islands Regency. Specifically, the objectives were (i) to perform morphological identification of captured commercial fish species; (ii) to determine the prevalence, intensity, and predilection sites of *Anisaki*s spp. infections; (iii) to conduct morphometric characterization of isolated larvae; and (iv) to undertake molecular identification and phylogenetic analysis of *Anisakis* spp. isolates, thereby clarifying their genetic relatedness to known regional populations. Through these integrated approaches, the study aimed to provide critical insights into the zoonotic and ecological threats posed by *A. typica*, informing future control strategies to safeguard fishery sustainability, marine ecosystem health, and public food safety.

## MATERIALS AND METHODS

### Ethical approval

Ethical approval for animal use was obtained from the Animal Ethics Committee, School of Veterinary Medicine and Biomedical Sciences, IPB University, Indonesia (Protocol No: 148/KEH/SKE/XII/2023).

### Study period and location

The research was conducted over 7 months, commencing with sampling activities in Sangihe Island Regency, North Sulawesi Province, Indonesia, from January to April 2024. This was followed by laboratory analyses, including morphological, morphometric, and molecular examinations, at the Helminthology Laboratory, IPB University, Bogor, Indonesia, from May to July 2024.

### Fish collection and morphological identification

**Figure 1:**
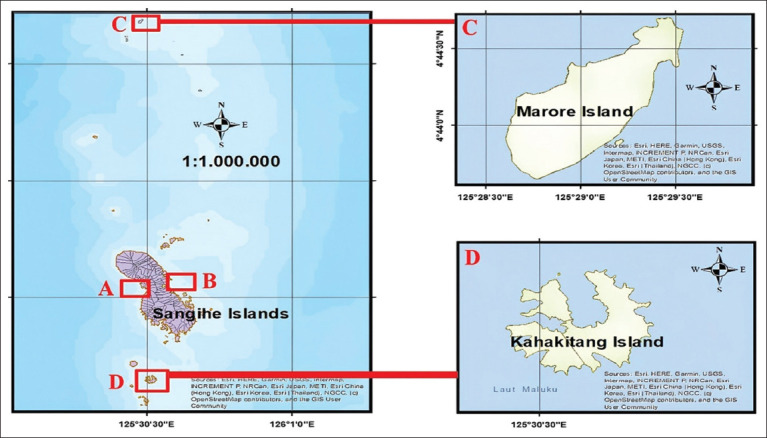
Map of research on the Sangihe Island cluster consisted of (a) Tahuna District and (b) North Tabukan District, the Marore Island cluster in (c) Marore District, and the Kahakitang Island cluster in (d) Tatoareng District.

The study was conducted across three geographi-cal clusters: The Sangihe cluster (Tahuna with 70 samples and North Tabukan Districts with 66 samples), the Kahakitang Island cluster (Tatoareng District with 20 samples), and the Marore Island cluster (Marore District with 22 samples), with precise coordinates provided for each sampling location (Figure 1). The sample size was determined using WinEpi 2.0 software (http://www.winepi.net/uk/index.htm) based on an unknown population, a 95% confidence level, an expected prevalence of 13.3%, and a 5% acceptable error margin [14]. The results of the sample calculations were as high as 178. The distribution of samples was executed in accordance with the habitat area and density-based proportional method, as detailed by Krebs [15], and included a modified diversity factor, as specified by Magurran [16]. Commercial fish samples were obtained from local fishermen between January and April 2024.

Fish samples were morphologically grouped based on body shape, coloration patterns, and standard length measurements. Samples were stored under refrigeration (approximately 4°C) and promptly transported to the Helminthology Laboratory of IPB University for parasitological examination through dissection. Before dissection, the standard length and total body weight of each fish specimen were recorded. The samples were further identified by morphometry to identify the fish [17].

### Parasite isolation and morphology

The digestive organs of each fish specimen were carefully dissected and examined visually and microscopically at appropriate magnifications. Larvae of *Anisakis* spp. were collected, enumerated, and preserved individually in 1.5 mL microcentrifuge tubes containing 70% ethanol. Larvae were morphologically identified based on coloration, size, anterior head morphology, gastrointestinal structures, and tail morphology [18–23] using an Olympus CX33 microscope (Japan) equipped with a digital camera at magnifications of 100× and 400×. Larval morphometric measurements were performed using ImageJ software. Representative *Anisakis* spp. larvae exhibiting similar morphological characteristics were selected for molecular characterization.

### Molecular identification and phylogenetic analysis

Morphologically similar *Anisakis* spp. larvae were selected for molecular identification. Preserved larval samples were washed thoroughly with phosphate-buffered saline to remove ethanol residues. Larvae were extracted using a Jetfllex™ Genomic DNA Purification Kit (Catalog Numbers A30700 and A30701, Thermo Fisher Scientific Inc., USA). The tissue lysis process was initiated by the addition of 20 μL of proteinase K, followed by an incubation period at 58°C for 1 h to ensure complete digestion of the tissue. Subsequently, RNase A was added, and the sample was incubated at 37°C for 5 min to remove any RNA contamination. The total duration of this stage varied from 1 h 15 min to 2 h, depending on the complexity of the sample. After lysis, genomic DNA (gDNA) was deposited, a step that took approximately 6–10 min. The deposited gDNA was then subjected to a washing procedure lasting 4–7 min to eliminate impurities. Finally, during the gDNA re-storage phase, samples were incubated at 50°C–55°C for 10 min to evaporate any residual ethanol, followed by a final incubation at 65°C for 1 h to ensure that the DNA was stable and dry.

Each polymerase chain reaction (PCR) was conducted in a 50 μL volume containing 2 μL of forward primer, 2 μL of reverse primer, 25 μL of MyTaq™ HS Red Mix (Geneaid Biotech Ltd., Taiwan), 16 μL of nuclease-free water, and 5 μL of DNA template. The PCR conditions followed the protocol described by Anshary *et al*. [24], with minor modifications, including increasing the reaction volume to 50 μL using the ready-to-use MyTaq HS Red Mix instead of separate components. Thermal conditions were adjusted by reducing the denaturation/elongation time to 15–30 s and increasing the annealing temperature to 56°C. In addition, primers were replaced to target the internal transcribed spacer (ITS) region of rDNA, thereby enhancing taxonomic resolution. The ITS region was identified as the optimal choice because of its superior resolution for differentiating species within the *Anisakidae* family. The ITS1–5.8S–ITS2 rDNA region (~965 bp) was amplified using the NC5 (5’-GTAGGTGAACCTGCGGAAGGATCATT-3’) with a concentration of 10 pmol/μL and NC2 (5’-TTAGTTTCTTTTTTCCTCCGCT-3’) with a concentration of 10 pmol/μL primers. PCR amplification was conducted under the following conditions: Initial denaturation at 95°C for 1 min; 35 cycles of denaturation at 95°C for 15 s; annealing at 56°C for 15 s; extension at 72°C for 10 s; and a final extension at 72°C for 5 min.

Forty microliters of the PCR product were subjected to amplification, and sequencing was performed by PT Genetika Science, Indonesia. Sequencing chromatograms were analyzed and aligned using MEGA11 software (Version 11.0.13, built for Windows ×64) (https://www.megasoftware.net/). The obtained nucleotide sequences were compared against the GenBank database using the Basic Local Alignment Search tool (http://www.ncbi.nlm.nih.gov/blast) to determine sequence similarity.

Phylogenetic relationships among *Anisakis* species were reconstructed using the neighbor-joining (NJ) method based on pairwise genetic distances. Phylogenetic reconstruction using NJ and pairwise distances was selected for suitability for displaying evolutionary proximity among similar species. *Anisakis* *simplex*, *Anisakis* *physeteris*, and *Anisakis* *pegreffii* were employed as outgroup taxa for phylogenetic tree construction. Genetic distances were computed using the pairwise distance method.

### Statistical analysis

The prevalence of parasitic infection was calculated as the number of infected fish divided by the total number of examined fish, multiplied by 100, and expressed as a percentage. The mean infection intensity was determined by dividing the total number of parasites by the number of infected fish hosts [25]. Fulton’s condition factor (K) was calculated using the following formula: K = (W/L³) × 100, where W is the body weight (g) and L is the standard length (cm) [26].

## RESULTS

### Identification of commercial fish

Twelve commercial fish species were identified among the 178 fish specimens examined. The most frequently encountered species in the waters bordering Indonesia and the Philippines were *Decapterus kurroides* (23%) and *Decapterus macrosoma* (21.9%). Based on their condition factor, these commercial fish demonstrated negative allometric growth patterns, indicating a disproportionate relationship between weight and length (average of condition factors: 0.87–1.42) (Table 1).

**Table 1:** Host and infection status of *Anisakis* spp. larvae.

Host	Length Mean ± SD	Weight Mean ± SD	NE	NI	*Anisakis* type 1	Prev (%)	Intensity	ACF
*Euthynnus affinis*	29.9 ± 11.3	481.5 ± 604.2	17	7	108	41.2	15.4	0.93
*Katsuwonus pelamis*	43.2 ± 7.9	1415 ± 821	13	5	39	38.5	7.8	1.12
*Decapterus kurroides*	20.2 ± 1.8	99.6 ± 26.8	41	4	23	9.8	5.8	0.87
*Variola louti*	20.8 ± 2.3	128.8 ± 61.4	21	3	4	14.3	1.3	0.96
*Decapterus macrosoma*	23.9 ± 3.4	137.7 ± 62	39	-	-	-	-	1.16
*Aprion virescens*	66.8 ± 4.5	3006.3 ± 374.3	4	-	-	-	-	0.99
*Ferdauia orthogrammus*	24.2 ± 14.2	373.3 ± 771.2	21	-	-	-	-	1.42
*Thunnus albacares*	57.6 ± 19.7	3516.8 ± 1236.4	6	-	-	-	-	1.04
*Gymnosarda unicolor*	58.7 ± 0.6	2354.3 ± 55.2	3	-	-	-	-	0.98
*Auxis rochei*	33.5 ± 17.8	845 ± 1206.4	5	-	-	-	-	1.00
*Elagatis bipinnulata*	46.3 ± 24.2	1352.3 ± 1232	3	-	-	-	-	1.24
*Lutjanus rufolineatus*	19.2 ± 1.6	93.3 ± 31.4	5	-	-	-	-	0.99
Total			178	19	174	10.7	9.2	

NE=Number examined, NI=Number infected, Prev=Prevalence, ACF: Average of condition factors

### Prevalence, intensity, and predilection

**Figure 2:**
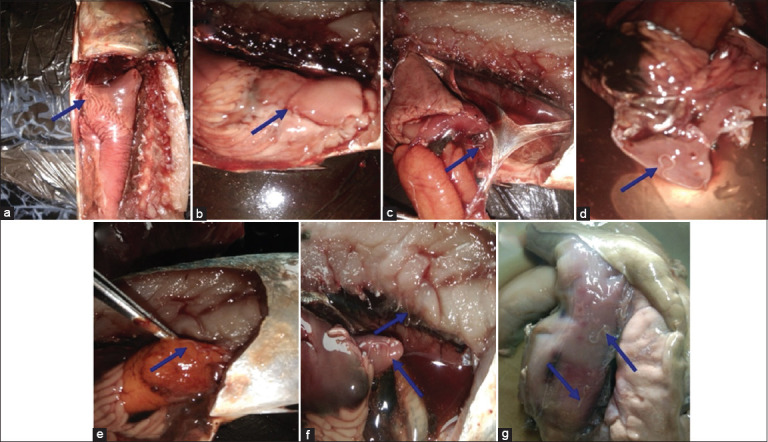
Predilection of *Anisakis typica (a) Pyloric ceca*, (b) Stomach wall, (c) Swim bladder, (d) Liver, (e) Gonad, (f) Muscle tissue, and (g) Intestinal wall.

Among the twelve identified species, four were found to be infected with *Anisakis* spp. The overall prevalence of *Anisakis* spp. infection in these four species was 10.7%. *Euthynnus affinis* emerged as the most susceptible host, displaying a prevalence of 41.2% and an infection intensity of 15.4 (Table 1). The intestinal wall was identified as the principal site of larval predilection for *Anisakis* spp. (Table 2 and Figure 2).

**Table 2:** *Anisakis* spp. larval morphometry (mean ± SD) and predilection percentages.

Host	BL (mm)	BT (mm)	V (mm)	M (mm)	Infected sites (%)							

					Stomach wall	Gonad	*Pyloric ceca*	Intestinal wall	Liver	Body cavity	Swim bladder	Muscle
*Euthynnus affinis*	15.69 ± 0.66	0.01 ± 0.00	0.43 ± 0.11	0.02 ± 0.01	1.9	0.9		96.3		0.9		
*Katsuwonus pelamis*	15.87 ± 0.91	0.01 ± 0.00	0.32 ± 0.15	0.02 ± 0.01				97.4		2.6		
*Decapterus kurroides*	12.82 ± 0.67	0.01 ± 0.00	0.59 ± 0.06	0.03 ± 0.00	9.2	40.9	13.6	13.6	9.2	4.5	4.5	4.5
*Variola louti*	15.59 ± 0.99	0.01 ± 0.00	0.44 ± 0.07	0.03 ± 0.00	25			75				

BL=Body length, BT=Boring tooth, V=Ventriculus, M=Mucron, SD=Standard deviation

### Spread of *Anisakis* spp.

**Figure 3:**
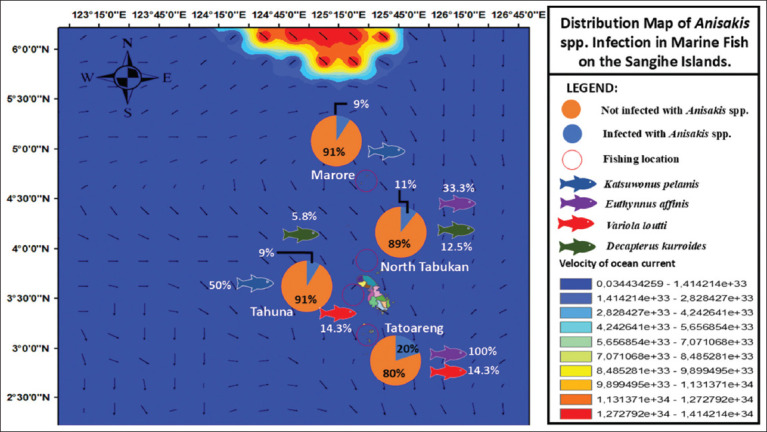
Distribution map of *Anisakis* spp. infection in marine fish based on the ocean current speed and direction.

The highest prevalence of *Anisakis* spp. infection was recorded in Tatoareng, reaching 20%. When assessed by fish species and location, *Katsuwonus pelamis* exhibited infection in Marore (28.6%) and Tahuna (50%). Infection in *E. affinis* was observed in North Tabukan (33.3%) and reached 100% in Tatoareng. *Anisakis* spp. infection in *D. kurroides* was found in North Tabukan (12.5%) and Tahuna (5.8%). In *Variola louti*, infections were documented in both Tahuna and Tatoareng, each at a prevalence of 14.3% (Figure 3).

### Morphology, morphometry, and molecular characterization

**Figure 4:**
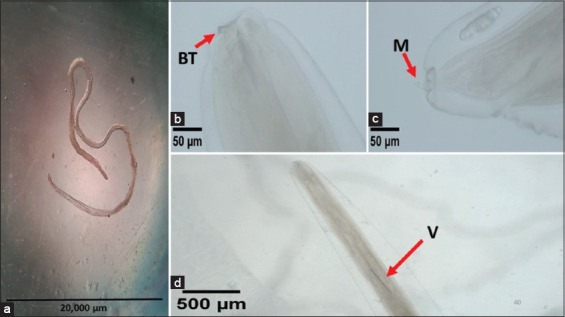
(a) *Anisakis* *typica*. (b) BT=Boring tooth 400× magnification, (c) M=Mucron 400× magnification, and (d) V=Ventriculus 100× magnification.

Morphological analysis classified the infective larvae as *Anisakis* type 1. Morphometric evaluation revealed a mean body length ranging from 12.82 to 15.87 mm, a boring tooth length of 0.01 mm, a ventriculus ranging from 0.32 to 0.59 mm, and a mucron length between 0.02 and 0.03 mm (Table 2 and Figure 4).

Molecular sequencing of the four *Anisakis* type 1 larvae collected from Sangihe confirmed their identity as *A. typica*. BLAST comparisons of nucleotide sequences at the ITS1–5.8S–ITS2 locus revealed varying degrees of genetic similarity: *A. typica* S2 (93.38%), S3 (98.84%), S4 (100%), and S5 (100%).

**Figure 5:**
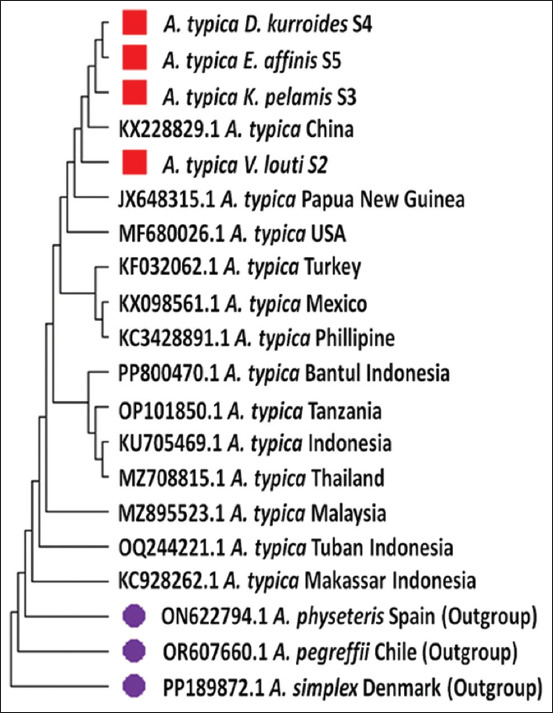
Phylogenetic tree of *Anisakis typica*. The red branch represents samples originating from the Western Pacific Ocean. This branch is clearly differentiated from samples of *A. typica* found in other countries (colorless) and from samples outside the group (purple), namely *Anisakis physeteris*, *Anisakis pegreffii*, and Anisakis *simplex*, which show different evolutionary relationships.

Phylogenetic analysis positioned the four *A. typica* isolates from Sangihe within a monophyletic clade alongside the *A. typica* reference sequence KX228829.1 from China. *A. simplex* was used as the outgroup for comparison (Figure 5). In terms of genetic proximity to the Chinese reference isolate, *A. typica* Sangihe 2 and 3 were most closely related, while Sangihe 4 and 5 exhibited greater divergence (Table 3).

**Table 3:** Proximity of *Anisakis* spp. sangihe sample to *A. typica* China.

Isolates	*A. typica* sangihe 2	*A. typica* sangihe 3	*A. typica* sangihe 4	*A. typica* sangihe 5	KX228829.1 *A. typica* China
*A. typica* sangihe 2					
*A. typica* sangihe 3	0.1465				
*A. typica* sangihe 4	0.2192	0.1535			
*A. typica* sangihe 5	0.6483	0.6365	0.5826		
KX228829.1 *A. typica* China	0.0000	0.0000	0.0776	0.4237	

*A. typica=Anisakis typica*

## DISCUSSION

### Ecological and economic relevance of fish species

This study identified 12 fish species in the Western Pacific Ocean, emphasizing their critical ecological roles and substantial economic value. These were primarily comprised of commercially important fish species used for human consumption and industrial applications [27]. Among these, tuna and skipjack are prominent export commodities that significantly contribute to Indonesia’s economy, with a combined export value of USD 865.73 million [28]. At the local level, the fishery sector contributes an average of 21.86% annually to the economic development of the Sangihe Regency [29]. However, economically important fish are also vulnerable to parasitic infections, which can affect their ecological roles and market value.

### Prevalence and regional comparison of *Anisakis* spp. infections

This study revealed that the total prevalence of *Anisakis* spp. infection among marine fish in the Western Pacific Ocean was 10.7%, with an intensity of 9.2. The observed prevalence corresponded to the classification for frequent, moderate-intensity infections [30]. The prevalence of *Anisakis* spp. in this study was lower than that recorded in other Indonesian waters, such as Banten (15%–90%), West Sulawesi (13.33%–30%), and East Java (17%) [31–33]. The observed prevalence was lower compared to neighboring regions such as the East China Sea (76.8%) [34], Australia (15%) [35], New Zealand (32%) [35], and the Philippines (22%) [36]; however, it exceeded that reported for Papua New Guinea (7.6%) [37].

### Growth patterns and climate-parasite interactions

Commercial fish species in the Western Pacific Ocean exhibit faster length growth relative to weight gain. This is in contrast to the findings for *E. affinis* in Malaysia, which reported a growth rate of <3 [38]. The same result was reported for *K. pelamis* in Pakistan, with negative allometric results [39]. This theory states that fish size reduction is influenced by climate change; that is, the oxygen supply for large fish cannot be fulfilled by their gills, whose surface area cannot meet the oxygen demand of their three-dimensional bodies [40]. In addition, it can be caused by parasitic infection of the fish body [41].

### Parasitic infection and ecosystem impacts

A high prevalence of parasitic infection contributes to growth impairment and affects ecosystem dynamics. Parasites can manipulate host behavior, thereby increasing predation susceptibility [42]. Such behav-ioral alterations may drive shifts in community composition and ecosystem dynamics. Furthermore, heavy parasitic infections negatively affected the growth and physiological conditions of the host fish. Studies have reported that as infection loads increase, the growth rate and overall condition of fish decline. When the energy intake of heavily infected fish is insufficient to meet their metabolic demands, they reach a critical threshold, which ultimately leads to mortality [43]. These interconnected effects demonstrate that parasitic infections affect not only individual hosts but also the broader ecosystem by altering energy flow, nutrient cycling, and predator-prey interactions.

### Host susceptibility and intermediate hosts

Based on the host, the fish species most suscepti-ble to infection by *Anisakis* spp. in the present study were *E. affinis*. *E. affinis*, commonly known as tongkol komo or the trade name of small tuna, belongs to the family Scombridae [44]. This fish is morphologically characterized by two or three dots between fin pairs [45]. Studies on the food preferences of this fish are described in the form of anchovies and small shrimp [46]. Another study by Vigneshwaran *et al*. [47] reported that this fish feeds on crustaceans, mollusks, and teleosts. Feeds such as mollusks and crustaceans (copepods, decapods, isopods, amphipods, and euphausiids) are intermediate hosts, where *Anisakis* spp. eggs develop into second-stage larvae [48].

### Environmental drivers of infection distribution

Environmental variables critically influence the prevalence and distribution of infections. The high prevalence of *Anisakis* spp. infection in Tatoareng (20%) compared to other locations may be explained by several environmental factors, such as fishing location (migration of definitive hosts, availability of intermediate hosts, and ocean currents) and fish size [49]. Although ocean currents at Tatoareng were weak, prevailing winds and currents directed toward the region (Figure 4), which may have been because intermediate hosts, such as crustaceans or predatory fish, brought more *Anisakis* spp. larvae to Tatoareng. Some of the factors that influence the high intensity of parasitic infections in fish are biotic factors, such as host age and size, parasite size, host specificity, season, host diet, host sex, and environmental factors [50].

### Migratory behavior and cross-regional spread

Three of the four fish species that were infected with *Anisakis* spp. were pelagic. These fish can migrate over long distances. Given the southward ocean current patterns (Figure 4), there is potential for fish to migrate toward the Makassar Strait. This is supported by studies stating that the skipjack habitat is in the southern Makassar Strait, with an optimum temperature of 28.78°C–31.25°C [51]. These fish may also migrate toward Java and Australian waters, increasing the potential for parasite dispersal across regions.

### Parasite localization and host-parasite dynamics

Predilection observations have shown that this parasite is found in digestive organs and the host body, particularly in *D. kurroides*. Dezfuli *et al*. [52] suggest that apart from *Pyloric ceca*, *Anisakis* spp. are microhabitats, especially in Salmon species. Larvae can migrate to the muscle of the fish both while the fish are alive and after death. The success rate of helminth infection depends largely on the ability of the helminth to manipulate or evade the common immune responses of the host [52]. The intestinal wall was identified as the principal site of predilection for the parasite. Studies have suggested that parasitic predilection can interfere with host movement and reduce swimming speed, acceleration, and turning ability, which in turn affect social interactions and group dynamics [53].

### Morphological differentiation of *Anisakis* type 1

The morphological identification of *Anisakis* spp. larvae was classified as type 1 based on the configuration of the boring tooth, structure of the ventriculus, and presence of a mucron at the tip of the tail [54]. The *Anisakis* type 1 group includes *A. simplex*, *A. pegreffii*, *A. typica*, *A. ziphidarum*, and *A. nascettii* [10]. A morphological examination revealed similar parasites. The key to its identification is the presence of a boring tooth, ventriculus, and mucron. The larval morphometric measurements corresponded closely with findings from previous studies on various hosts in Vietnam and Thailand, such as ventriculus length 0.48–0.94 mm and mucron length 0.01–0.03 mm [55, 56]. These findings highlight the importance of host and parasite morphological traits in determining host specificity and infection outcomes.

### Molecular confirmation and novel hosts

The molecular identification results indicated that all *Anisakis* spp. in this study were *A. typica*. This parasite was found in Scombridae (*E. affinis* and *K. pelamis*), Carangidae (*D. kurroides*), and *Serranidae* (*V. louti*). The results of this study were corroborated by data showing that *K. pelamis* in South Sulawesi, Japan, Hawaii, and Moorea is also infected with *A. typica* [9, 24, 57]. To the best of our knowledge, this is the first documentation of *A. typica* infection in *D. kurroides* and *V. louti*. *A. typica* has only been reported to infect *D. macrosoma*, *D. maruadsi*, *D. tabl* [36], *D. macarellus* [55], and *D. russelli* [58]. Palm *et al*. [59] have also mentioned that *D. kurroides* is infected with *Anisakis* spp. but did not specifically mention the *Anisakis* species.

### Phylogenetic insights and genetic variation

Based on its evolutionary development, the phylogenetic tree of *A. typica* from the Western Pacific Ocean was derived from a common ancestor from China (KX228829.1) and Papua New Guinea (JX648315.1). These phylogenetic results were similar to those of *A. typica* in Vietnam, which is adjacent to Papua New Guinea (JX648312) [54]. Phylogenetic analysis revealed genetic variation in the *A. typica* population along the Western Pacific Ocean. Genetic variations are caused by several factors. The first factor is the occurrence of DNA mutations, the second is gene flow due to genetic exchange from one population to another, the third factor is the occurrence of genetic recombinants, and the fourth factor is natural selection as an adaptive effort of an organism [60].

### Biogeography and host migration

Based on proximity, this study provides important implications, namely the implication of high genetic similarity between samples of *A. typica* Sangihe 2 and Sangihe 3 and *A. typica* from China. This suggests the possibility of a historical host migration of this parasite. These findings underscore the necessity of further research to monitor definitive host migration pathways and develop effective control measures along the Indonesian-Philippine border. This is similar to a study that revealed the zoogeographical distribution of *A. typica* in Brazil, which mentioned that the definitive hosts of this parasite are *Peponocephala electra*, *Stenella clymene*, and *Kogia breviceps*, with a distribution area on the Atlantic coast of Brazil [61].

### Role of marine mammals as definitive hosts

This parasite’s transmission is facilitated through definitive hosts, primarily dolphins [62]. Manoppo *et al*. [63] mentioned that there are marine mammals that have been encountered by the Sangihe Islands Regency Community, such as Dugong and Whales in the North Tabukan district and dolphins in the Kendahe and Manganitu districts. Consequently, dolphins and whales are suspected to act as definitive hosts facilitating *A. typica* transmission along the Indonesia-Philippines border. This was reinforced by the identification of *A. typica* in pygmy sperm whales in the Southern Philippines [64].

### Significance of *A. typica* in *V. louti* and coral reef fisheries

An interesting finding of this study is the infec-tion of *V. louti* with *A. typica*. This fish species belongs to a group of shallow-water groupers that inhabit coral reef habitats at depths of 15–250 m [65]. This species is often caught by fishers and is abundant along the Indo-Pacific border. This is because local fishermen in the Sangihe Islands Regency generally have small boats and traditional fishing gear, which causes fishing activities to be concentrated more in coral reef areas [66]. This species demonstrates relatively rapid growth and early maturity, making it less susceptible to overexploitation than other groupers [65]. Studies by Justine [67] and Bray and Justine [68] reported parasitic infections by these species, including those of *Cucullanus* spp. and *Digenea*. This finding warrants serious consideration given the potential for *Anisakis* spp. infection to cause anisakiasis through the consumption of raw or improperly processed fish and cause allergic reactions in humans if not properly cleaned by removing the parasite due to allergenic substances still attached to the fish when processed or consumed [69].

### Public health risks and zoonotic potential

*A. typica* poses major risks to public health and ecosystem sustainability. Experimental studies have suggested that *A. typica* can infect mice after observation for 2–8 h. In experiments using mice, we found that mice experienced moderate-to-severe inflammation. Inflammation is evidenced by mucosal erosion and ulceration and the presence of polymorphonuclear cells [70]. Based on these findings, *A. typica* is considered to have zoonotic potential. This is because mice have 12% less genome than humans, which means that mice have similarities with humans and have implications for modeling human diseases [71, 72].

### Recommendations for control and prevention

Visual inspection of parasites is essential not only to avoid contamination but also to reduce their ecological impact. Improper handling of infected fish or discarding offal containing *Anisakis* spp. larvae from the sea can increase environmental contamination, potentially facilitating the spread of the parasite within marine ecosystems [73]. Limiting the spread of parasites requires actions such as wearing gloves when cleaning fish and ensuring that offal is properly disposed of. *Anisakis* spp. not only cause anisakiasis but can also cause allergic reactions [74]. This preventive measure not only safeguards human health but also contributes to the ecological balance by preventing the accidental spread of parasitic diseases in marine environments.

## CONCLUSION

This study provides the first integrated report on the occurrence, genetic characterization, and ecological implications of *A. typica* infection in commercial fish species from the Western Pacific Ocean, particularly in the Indonesian–Philippines maritime zone. Among 178 fish specimens across 12 species, *A. typica* was identified in four species, with an overall infection prevalence of 10.7% and moderate infection intensity (mean intensity: 9.2). *E. affinis* exhibited the highest susceptibility (41.2%, intensity: 15.4), with the intestinal wall being the principal predilection site. Molecular and phylogenetic analyses confirmed the identity of *A. typica* and revealed a close genetic relationship with isolates from China, suggesting possible host migration routes.

The study holds significant practical implications for fisheries management and food safety. The presence of *A. typica* in economically important fish such as *E. affinis*, *K. pelamis*, *D. kurroides*, and *V. louti* raises concerns regarding zoonotic transmission risks, export market integrity, and consumer health, particularly given the potential for anisakiasis and allergic reactions. These findings underscore the need for strengthened surveillance, hygienic handling practices, and routine parasitological screening in fish destined for human consumption and export.

The strengths of this study lie in its combined application of morphological, morphometric, and molecular methods, its coverage of under-reported geographic regions, and the novel documentation of *A. typica* in *D. kurroides* and *V. louti*. In addition, the ecological analysis provides insight into environmental drivers influencing parasite prevalence and distribution.

However, the study is not without limitations. The cross-sectional nature of the sampling limits temporal inference, and the sample size, although statistically sufficient, may not capture broader seasonal or species-specific variability. Moreover, the absence of data on definitive marine mammal hosts in the region restricts understanding of the complete life cycle of *A. typica* in this ecosystem.

For future research, longitudinal studies are needed to monitor infection dynamics over time and across migratory patterns. Investigations into allergenic protein persistence, host immune responses, and the role of marine mammals as definitive hosts would further elucidate the zoonotic potential of *A. typica*. Expansion to additional coastal regions and integration with public health data would also strengthen One Health applications.

In summary, this study highlights the ecological relevance, zoonotic potential, and transboundary distribution of *A. typica* in commercially important fish species. It advocates for a multidisciplinary, One Health-based surveillance framework to mitigate parasitic threats to marine ecosystems, food safety, and human health.

## AUTHORS’ CONTRIBUTIONS

FS, SS, RT, and ABN: Conceptualized and designed the study. DDP: Sample collection. FS, SS, and DDP: Statistical analysis and interpreted the data. SS, RT, and ABN: Molecular and morphological identification. DDP: Drafted and revised the manuscript. All authors have read and approved the final manuscript.
